# Pharmacokinetics of the Novel Echinocandin CD101 in Multiple Animal Species

**DOI:** 10.1128/AAC.01626-16

**Published:** 2017-03-24

**Authors:** Voon Ong, Kenneth D. James, Steven Smith, B. Radha Krishnan

**Affiliations:** a Cidara Therapeutics, Inc., San Diego, California, USA; b Seachaid Pharmaceuticals, Durham, North Carolina, USA

**Keywords:** CD101, Candida, antifungal agents, echinocandin, pharmacokinetics, preclinical drug studies

## Abstract

CD101 is a novel semisynthetic echinocandin with antifungal activity against Candida and Aspergillus spp. The pharmacokinetics (PK) of CD101 administered intravenously to mice, rats, dogs, cynomolgus monkeys, and chimpanzees are presented. CD101 consistently exhibited very low clearance, a modest volume of distribution at steady state (*V*_ss_), and a long half-life (*t*_1/2_) across all species tested. In mouse, rat, dog, cynomolgus monkey, and chimpanzee, CD101 clearance was 0.10, 0.47, 0.30, 0.41, and 0.06 ml/min/kg, respectively; *V*_ss_ was 206, 1,390, not determined, 597, and 400 ml/kg, respectively; and *t*_1/2_ was 25, 39, 53, 40, and 81 h, respectively. CD101 demonstrated a lower clearance and correspondingly longer half-life than those of anidulafungin, with more pronounced differences in higher species (anidulafungin *t*_1/2_, 8 h in cynomolgus monkey and 30 h in chimpanzee). In the rat, tissue/plasma area under the concentration-time curve (AUC) ratios, in descending order, were 4.62 (kidney), 4.33 (lung), 4.14 (liver), 3.87 (spleen), 1.09 (heart), and 0.609 (brain), indicating that CD101 exposure relative to plasma levels was comparable for major organs (approximately 4-fold higher in tissue than in plasma), with the exception of the heart and brain. Biliary elimination of intact CD101 was the predominant route of excretion; the mean cumulative amount of CD101 excreted into the bile and feces over the course of 5 days accounted for 22.6% and 27.7% of the total dose administered, respectively. There were no sex differences in the pharmacokinetics of CD101. Given its low clearance, long half-life, and wide tissue distribution, CD101 once weekly is expected to provide appropriate systemic levels for treatment and prevention of invasive fungal infections.

## INTRODUCTION

Echinocandins are used to treat serious invasive fungal infections and are recommended for first-line treatment of suspected or confirmed candidemia and invasive Candida infections (1). Overall, echinocandins are well tolerated, have low drug interaction potential, have been shown to be safe in animal toxicology and reproductive development studies, and have been safely used for over 15 years (2, 3). Yet there are unmet needs with the currently available echinocandins (anidulafungin [Pfizer, New York, NY], caspofungin [Merck, Whitehouse Station, NJ], and micafungin [Astellas Pharma, Northbrook, IL]), which are approved only for once-daily intravenous (i.v.) administration. This dosing regimen not only is difficult to maintain beyond the inpatient setting but may also provide inadequate pharmacokinetic (PK)/pharmacodynamic exposure for efficacy, particularly against increasingly prevalent non-albicans Candida (4, 5).

CD101, a novel echinocandin, is in development as an i.v. formulation for the treatment and prevention of serious systemic fungal infections. Consistent with the echinocandin class, CD101 inhibits the synthesis of 1,3-β-d-glucan, has excellent *in vitro* activity against a broad spectrum of clinically important Candida spp., including emerging pathogens of concern, such as azole-resistant Candida glabrata (6, 15; D. Hall, R. Bonifas, L. Stapert, M. Thwaites, D. L. Shinabarger, and C. M. Pillar, submitted for publication), and demonstrates a concentration-dependent antifungal effect. CD101 has shown a strong correlation between both area under the curve (AUC)/MIC and maximum concentration of drug (*C*_max_)/MIC ratios and fungicidal activity (coefficient of determination of 0.905 and 0.907, respectively) (C. M. Rubino, V. Ong, D. Thye, and P. G. Ambrose, presented at the 55th Interscience Conference of Antimicrobial Agents and Chemotherapy/28th International Congress of Chemotherapy Joint Meeting, San Diego, CA, 17 to 21 September 2015; E. A. Lakota, C. M. Rubino, V. Ong, K. Bartizal, L. Miesel, S. M. Bhavnani, and P. G. Ambrose, presented at ASM Microbe, Boston, MA, 16 to 20 June 2016) and has demonstrated *in vivo* efficacy in mouse models of disseminated Candida and Aspergillus infection (7, 16). Unlike currently approved once-daily, IV-only echinocandins, CD101 is highly stable, enabling additional formulations and potential uses. In preclinical and phase 1 studies conducted to date, CD101 has demonstrated distinctive PK characteristics, notably the ability to safely achieve high plasma concentrations and an exceptionally long half-life (*t*_1/2_) (9, 10).

A series of preclinical studies was conducted to evaluate the PK profile of CD101 in multiple species, including nonhuman primates, and to facilitate projection of human PK.

(Results reported here were presented in part at the 54th Interscience Conference on Antimicrobial Agents and Chemotherapy [ICAAC], Washington, DC, 5 to 9 September 2014, and at the 26th European Congress of Clinical Microbiology and Infectious Diseases [ECCMID], Amsterdam, the Netherlands, 9 to 12 April 2016.)

## RESULTS

### Animal pharmacokinetics.

**FIG 1:**
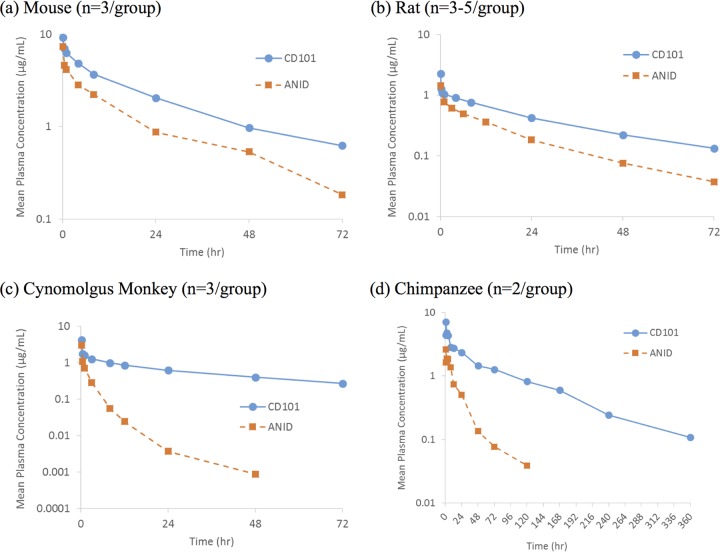
Mean concentration-time profiles in plasma following intravenous (normalized to 1 mg/kg) administration of CD101 or anidulafungin (ANID) in mouse, rat, cynomolgus monkey, and chimpanzee.

The PK profiles of CD101 i.v. administration in mice, rats, dogs, cynomolgus monkeys, and chimpanzees are shown in Table 1, as are the results following intraperitoneal (i.p.) CD101 administration to mice. Following i.v. administration, CD101 consistently exhibited a favorable (i.e., linear) PK profile, namely, very low clearance, modest volume of distribution, and long half-life (Table 1) across all species tested. Exposures, as measured by *C*_max_ and AUC, showed dose-dependent increases and were generally dose proportional. No PK differences between males and females were found with CD101 in rats (Table 1) or monkeys (data not shown). Comparisons were made with anidulafungin (i.v. only) as anidulafungin displayed the lowest clearance and longest *t*_1/2_ of the three currently approved echinocandins. CD101 displayed a long half-life and low clearance relative to the values for anidulafungin (Fig. 1), with a trend toward more pronounced differences in higher species (i.e., cynomolgus monkey and chimpanzee). The *t*_1/2_ and clearance in the mouse were 25 h and 0.10 ml/min/kg for CD101 and 19 h and 0.21 ml/min/kg for anidulafungin, respectively. Differences were more pronounced in nonhuman primates. For cynomolgus monkeys, the *t*_1/2_ and clearance were 40 h and 0.41 ml/min/kg for CD101 and 8 h and 5.03 ml/hour/kg for anidulafungin, respectively; for chimpanzee, the CD101 *t*_1/2_ was 2.7-fold longer (81 versus 30 h), and clearance was over 7-fold lower (0.06 ml/min/kg versus 0.42 ml/min/kg) than the values for anidulafungin. It should be noted that the chimpanzee PK data were determined from only two animals due to the limited availability of this species for research.

**TABLE 1:** CD101 pharmacokinetic parameters following intravenous administration to mouse, rat, dog, monkey, and chimpanzee and intraperitoneal administration to mouse

Animal model and route^*a*^	Dose (mg/kg)	Sex	*T*_max_ (h)	*C*_max_ (μg/ml)	AUC_0–*t*_ (μg · h/ml)	AUC_0-inf_ (μg · h/ml)	*t*_1/2_ (h)	CL (ml/min/kg)^*c*^	*V*_ss_ (ml/kg)^*d*^
Mouse									
i.v. bolus	1	Male	0.083	9.29	87.3	166	25.2	0.102	206
i.p.	1	Female	1.00	3.97	59.6	88.9	71.1	ND	ND
	4	Female	1.00	13.6	245	313	44.5	ND	ND
	16	Female	1.67	52.0	902	1200	60.8	ND	ND
Rat									
i.v. bolus	5	Female	0.083	10.5	185	188	35.7	0.444	1210
		Male	0.083	12.0	180	183	34.9	0.456	1160
	15	Female	0.083	27.6	435	441	34.9	0.567	1450
		Male	0.083	35.9	562	580	42.7	0.431	1420
	45	Female	0.083	115	1490	1510	39.9	0.496	1550
		Male	0.083	164	1610	1660	44.5	0.452	1570
Dog									
i.v. bolus	1.4	Male	0.083	1.57	48.7	ND^*b*^	53.1	0.301	ND
Cynomolgus monkey									
i.v. bolus	2.13	Male	0.167	9.07	92.1	ND	39.7	0.405	597
Chimpanzee									
i.v. infusion	1	Female	1.00	7.15	290	302	80.7	0.0568	400

a i.p., intraperitoneal; i.v., intravenous.

b ND, not determined.

c CL, clearance.

d *V*_ss_, volume of distribution at steady state.

### Tissue distribution.

**FIG 2:**
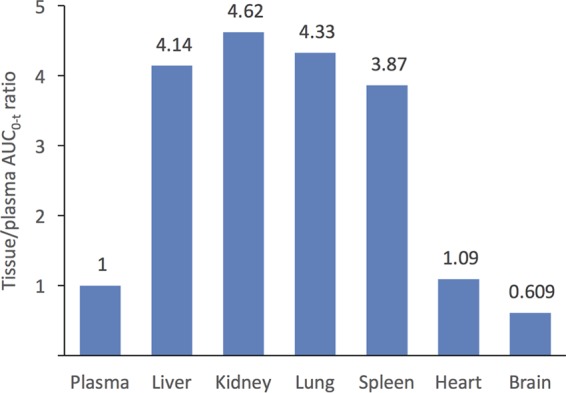
Tissue/plasma AUC_0–*t*_ ratio of CD101 following intravenous administration of 5 mg/kg in rats.

For each tissue as well as plasma sample, the AUC was calculated as it would better represent a time-averaged profile of exposures for comparison as opposed to point estimates, which may be affected by differing rates of elimination from each organ. Tissue/plasma AUC ratios in rats following i.v. administration indicated that CD101 exposures relative to plasma were comparable for highly perfused major organs, with the exception of the heart and brain (Fig. 2). Mean AUC_0–*t*_ (where *t* = 120 h) values ranged from 1,100 μg · hr/g in kidney tissue to 145 μg · hr/g in brain tissue. Tissue/plasma AUC ratios were approximately 4- to 5-fold higher in kidney, lung, liver, and spleen than in plasma (Fig. 2). Maximum plasma concentrations (*C*_max_) of CD101 were observed at the first sampling time (5 min) for plasma and tissues, with the exception of heart (time to maximum concentration of drug [*T*_max_] = 8 h) and brain (*T*_max_ = 48 h) tissue.

### Excretion.

**FIG 3:**
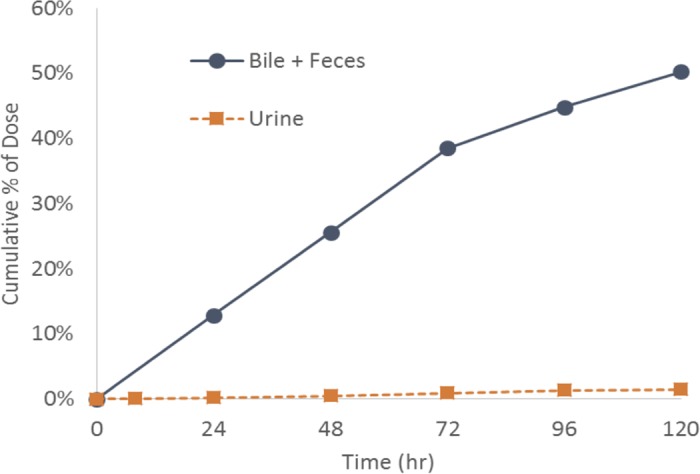
Mean cumulative CD101 as a percentage of dose measured in excreta following intravenous administration of 5 mg/kg in rats.

Figure 3 shows the mean cumulative amount of CD101 as a percentage of dose, measured in excreta following a single 5-mg/kg dose of CD101 i.v. in bile duct-cannulated rats. Biliary elimination of CD101 as intact drug into bile/feces was the predominant route of excretion; the mean cumulative amount of CD101 excreted into the bile and feces over the course of 5 days accounted for 22.6% and 27.7% of the total dose administered, respectively. Only 1.4% of the total dose was recovered in the urine during that same interval.

## DISCUSSION

The PK profile of CD101 across all animal species tested (mice, rats, dogs, and nonhuman primates) consistently exhibited a long half-life (i.e., low clearance) following i.v. administration, with a half-life of 81 h in the chimpanzee following a 1-mg/kg dose. Data in the chimpanzee can provide interesting insight for projection and comparison with human PK. Following the precedent set by Hadju and colleagues (11) for use of the chimpanzee as a surrogate model of human PK during product candidate selection, which ultimately led to development of caspofungin, we also compared chimpanzee and human PK profiles of anidulafungin and found remarkable similarity (12). It was anticipated that CD101 PK in the chimpanzee also would be predictive of its human PK based on the similar between-species protein binding and body weights used in allometric scaling and comparisons, and, indeed, the chimpanzee as a surrogate model for CD101 human PK was superior to allometric scaling for estimation of human clearance/volume of distribution and predicted half-life (12). Although it was apparent that clearance of CD101 was anticipated to be very low, allometric scaling was unable to predict the multiphasic PK profile observed in the chimpanzee or human PK profile.

CD101 has demonstrated a longer half-life than the currently approved echinocandins, and since of these anidulafungin is known to possess the lowest clearance/longest half-life (12), anidulafungin was selected as the reference compound/comparator for the various studies of CD101 animal PK. The half-life of CD101 in human is about 3-fold longer (about 90 h) than that of anidulafungin (about 30 h) (10). It should be noted that 90 h is the effective half-life of CD101 (i.e., the half-life that covers the majority of the AUC), with a longer, later-phase half-life evident at lower concentrations beyond the first week after dosing. The effective half-life and related AUC of CD101 suggest that CD101 can be administered i.v. once weekly (Lakota et al., presented at ASM Microbe, 2016), potentially enabling earlier hospital discharges and use of CD101 in situations in which the currently available echinocandins would not be considered due to the impracticalities of daily infusion (e.g., outpatient therapy).

The distribution of CD101 in tissue as evaluated in rats showed that the mean AUC_0–*t*_ was lowest in brain tissue and highest in kidney tissue. Calculation of tissue/plasma AUC ratios indicated that exposure relative to plasma was comparable for major organs, with the exception of notably lower ratios in heart and brain tissues. In the case of brain concentrations, there was little difference across all the collection intervals. Given the relatively low concentrations and small differences across times, measured concentrations may reflect contamination from residual whole blood during the collection process as organs were harvested without prior perfusion. Regardless, the passage of CD101 across the blood-brain barrier appears to be very low. The tissue distribution of CD101 appears similar to that of anidulafungin in terms of having fairly constant levels across major organs of elimination. The tissue/plasma ratio for CD101 in the liver was lower than that of other echinocandins, which, together with the lack of toxic/reactive intermediates, may contribute to the lack of hepatotoxicity with CD101 (9). CD101 has also been shown to be highly protein bound, similar to anidulafungin, and consistent between animal species and human plasma (from 97.8% to 99.1% in CD-1 mouse, Sprague-Dawley rat, cynomolgus monkey, and chimpanzee and 98.7% in human) (9). The relationship between echinocandin distribution and efficacy and the clinical relevance of differences in tissue levels remain equivocal. As noted by Damle et al. (13), who reported the higher volume of distribution of anidulafungin than the values for caspofungin and micafungin, the proportion of drug available for antifungal activity (i.e., unbound versus bound drug) was not considered by previous studies. The relatively lower tissue penetration of caspofungin and micafungin than that of anidulafungin (Table 2) does not correlate with reduction in fungal tissue burden observed in animal models of fungal infection. In fact, caspofungin, despite having the lowest tissue/plasma exposure ratio in the lung (1.2), is the only echinocandin among those currently available that is approved for treatment of pleural space infections although this distinction may be more a factor of the indications pursued during development than of the relative efficacies of the compounds.

**TABLE 2:** Comparative tissue/plasma AUC exposure ratios for CD101 and marketed echinocandins

Tissue	Tissue/plasma AUC ratio^*a*^			
	CD101	CASP	MICA	ANID
Liver	4.14	10.2	7.8	12.4
Kidney	4.62	5.5	3.2	10.7
Lung	4.33	1.2	3.6	10.4

a Values for caspofungin (CASP), micafungin (MICA), and anidulafungin (ANID) are from reference 13.

The excretion of CD101 in the bile/feces (∼50%) is highly comparable to that of anidulafungin as reported by Damle and colleagues (14). In that study, which utilized radiolabeled anidulafungin in bile duct-cannulated rats, 94.8% of the radioactive dose was accounted for through 168 h (or 7 days). Drug-derived radioactivity was recovered in the carcass, bile, and feces, accounting for 40.4%, 33.9%, and 17.1% of the dose administered, respectively. Only 2.94% of the radioactivity was recovered in the urine. In a similar study of CD101 conducted with nonradiolabeled material, no biotransformation was observed for CD101 in plasma, bile samples, and excreta collected (data not shown). This is also consistent with results of *in vitro* metabolite profiling conducted previously using liver microsomes or hepatocytes (9).

CD101's concentration-dependent pattern of fungicidal activity (7, 8), in combination with its slow clearance from the body, has important implications for dose regimen selection and front-loading drug exposure (i.e., maximizing drug effect early in the course of therapy to increase the rate and extent of pathogen killing, reduce and prevent resistance, and ultimately improve clinical outcomes), as front-loading drug exposure is most beneficial with antimicrobial agents such as CD101 that have a concentration-dependent effect and long half-life.

In this series of PK studies, CD101 consistently exhibited a favorable linear PK profile across all species, mainly attributable to very low clearance resulting in a longer half-life. Additionally, there was little to no drug accumulation of CD101 and no sex-based differences (in the rat and monkey) after multiple doses. These data support the characterization of CD101 as a novel echinocandin candidate for the treatment of serious, life-threatening, invasive fungal infections.

## MATERIALS AND METHODS

All studies that involved animals adhered to the International Guiding Principles for Biomedical Research Involving Animals, as revised by the International Council for Laboratory Animal Science (ICLAS) and the Councils for International Organizations of Medical Sciences (CIOMS) in 2012.

### Echinocandin compounds.

CD101 was prepared by Cidara Therapeutics, Inc. (San Diego, CA). Anidulafungin, obtained commercially (Molcan, Toronto, Canada), was used as a comparator in subsequent studies (i.v. only, at lowest dose). Dosing solutions were prepared on the day of dosing by accurate weighing of compound into appropriately sized containers and formulated in a vehicle consisting of 0.9% saline with 1% polysorbate (Tween) 20. Stock solutions for bioanalysis were prepared as 10-mg/ml solutions in dimethyl sulfoxide (DMSO) with subsequent serial dilutions into methanol-water (1:1) working solutions.

### Dose selection in different species.

Selection of doses for the species involved in these studies followed the general principle of allometric scaling, i.e., lower doses for larger species. Intravenous administration was the main route of administration, and, to avoid enzyme saturation/inhibition that may lead to nonlinear kinetics, doses were kept lower (1 mg/kg), particularly for i.v. bolus administration. Intraperitoneal administration in the mouse was also reported for comparison with i.v. and to provide an estimate of exposure and bioavailability following i.p. administration, which is the typical, preferred route for infection models. Higher CD101 dose ranges were provided for the mouse and rat to demonstrate dose linearity/proportionality.

### Pharmacokinetics. (i) Mice.

A single 1-mg/kg dose of CD101 or anidulafungin was administered by slow i.v. bolus (∼2 min) to male ICR (CD-1) mice (*n* = 3/group/time point). Blood samples (via cardiac puncture) were collected for plasma processing predose and at 0.083 (5 min), 0.5, 1, 4, 8, 24, 48, and 72 h postdose. PK profiles were generated based on mean results from the three animals sampled at each time point. In another study, a single 1-, 4-, or 16-mg/kg dose of CD101 was administered by i.p. injection to female ICR (CD-1) mice (*n* = 3/time point/dose), and blood samples (via cardiac puncture) were drawn for plasma processing at 1, 3, 6, 12, 24, 48, 72, and 96 h postdose.

### (ii) Rats.

In one of three rat studies reported here, a single 5-, 15-, or 45-mg/kg dose of CD101 was administered by slow i.v. bolus (∼3 min) to Sprague-Dawley rats (*n* = 3 to 5/dose). Blood samples (via jugular vein catheter [JVC]) were drawn for plasma processing at 0.083, 0.25, 0.5, 1, 2, 4, 8, 24, 48, 120, 168, and 240 h after dosing. In the second rat study, conducted to assess tissue distribution, a single 5-mg/kg dose of CD101 was administered by slow i.v. bolus (∼3 min) to male Sprague-Dawley rats (*n* = 3 animals/time point). Blood samples (via cardiac puncture) were collected for plasma processing predose and at 0.083, 0.5, 1, 2, 4, 8, 24, 48, 72, 96, and 120 h postdose. In parallel with blood collection, animals were euthanized, and tissues (liver, lungs, kidneys, heart, spleen, and brain) were excised and frozen prior to analysis. In the third rat study, conducted to assess excretion, a single 5-mg/kg dose of CD101 was administered by slow i.v. bolus (∼3 min) to bile duct-cannulated male Sprague-Dawley rats (*n* = 3). Bile samples were collected from each animal into prelabeled bile collection tubes predose and at 0 to 8, 8 to 24, 24 to 48, 48 to 72, 72 to 96, and 96 to 120 h postdose; urine samples were collected from each animal at the same time points pre- and postdose.

### (iii) Dogs.

A single 1.4-mg/kg dose of CD101 was administered by slow i.v. bolus (∼10 min) to male beagle dogs (*n* = 4), and blood samples (via jugular vein) were drawn for plasma processing at 0.083, 0.33, 0.75, 1.5, 4.5, 12, 24, 48, and 72 h postdose.

### (iv) Monkeys.

A single dose of 2.13 mg/kg CD101 or 2.8 mg/kg anidulafungin was administered by slow i.v. bolus (∼10 min) to cynomolgus monkeys (*n* = 3/group), and blood samples (via peripheral vein) were drawn for plasma processing at 0.167, 0.5, 1, 3, 8, 12, 24, 48, and 72 h postdose.

### (v) Chimpanzees.

A single 1-mg/kg dose of CD101 or anidulafungin was administered by i.v. infusion (1 h) to female chimpanzees (*n* = 2/group), and blood samples (via peripheral vein) were drawn for plasma processing at the midpoint of infusion, at the end of infusion, at 2, 4, 8, 12, 24, 48, and 72 h postdose, and on days 5, 7, and 10 postdose.

### Plasma and tissue sample processing and analysis.

For each of the pharmacokinetic studies, whole-blood samples (K_3_EDTA as anticoagulant) were collected and centrifuged within 30 min of collection. The resulting plasma was harvested and stored at −20°C until analysis. Tissue samples were homogenized by a bead-based homogenizer following the addition of deionized water to excised tissue in a 2:1 (vol/wt) ratio and stored at −20°C until analysis. Prior to analysis, plasma or homogenized tissue samples were quenched with acetonitrile (3:1, acetonitrile-plasma or tissue ratio) containing an appropriate internal standard (isotopically labeled d9-CD101 [mouse and rat] or a structural analog [dog, monkey, and chimpanzee]). Plasma and tissue homogenate concentrations (quantified against calibration standards processed the same way) were determined by reverse-phase, gradient liquid chromatography with tandem mass spectrometric detection (LC-MS/MS) using an AB-SCIEX API 3200Q/4000 mass spectrometry system. Calibration standard ranges were the following: 0.400 (lower limit of quantitation, [LLOQ]) to 200 μg/ml (CD101, mouse), 0.040 (LLOQ) to 10 μg/ml (anidulafungin, mouse), 0.002 (LLOQ) to 10 μg/ml (CD101, rat), 0.005 (LLOQ) to 10 μg/ml (anidulafungin, rat), 0.050 (LLOQ) to 10 μg/ml (CD101, dog), 0.020 (LLOQ) to 10 μg/ml (anidulafungin, dog), 0.010 (LLOQ) to 10 μg/ml (CD101, monkey), 0.001 (LLOQ) to 10 μg/ml (anidulafungin, monkey), 0.015 (LLOQ) to 10 μg/ml (CD101, chimpanzee), and 0.015 (LLOQ) to 10 μg/ml (anidulafungin, chimpanzee). When necessary, dilutions with blank matrix were made for samples that quantified above the upper limit of quantitation. The methods used in the analysis were qualified as fit for purpose: calibration standards were matrix matched with the exception that human plasma calibration standards were used to analyze chimpanzee plasma samples due to the rarity of blank chimpanzee plasma, and, for each analytical batch, triplicate calibration standards were included at the beginning, approximately in the middle, and at the end of the batch. Following analysis, quantitation was carried out by a calibration curve comprising the analyte/internal standard area ratio versus concentration. Analytical batch acceptance followed the general guidance that triplicate standards/quality controls be within ±20% for accuracy (percentage of nominal concentration) and ≤20% for precision (percent coefficient of variation).

### Pharmacokinetic data analysis.

Pharmacokinetic parameters were calculated by noncompartmental analysis using Phoenix WinNonlin (version 6.3; Pharsight, Mountain View, CA) from either individual or mean (mouse) concentration-time profiles. An unpaired *t* test was used to compare mean exposures from male and female rats.
